# COVID-19 Vaccine Equity and Access: Case Study for Health Care Chatbots

**DOI:** 10.2196/39045

**Published:** 2023-01-25

**Authors:** Jose G Perez-Ramos, Mariela Leon-Thomas, Sabrina L Smith, Laura Silverman, Claudia Perez-Torres, Wyatte C Hall, Suzannah Iadarola

**Affiliations:** 1 Department of Public Health Sciences School of Medicine and Dentistry University of Rochester Rochester, NY United States; 2 Department of Pediatrics University of Rochester Medical Center Rochester, NY United States

**Keywords:** mHealth, ICT, Information and Communication Technology, community, chatbot, COVID-19, health equity, mobile health, health outcome, health disparity, minority population, health care gap, chatbot tool, user experience, chatbot development, health information

## Abstract

**Background:**

Disparities in COVID-19 information and vaccine access have emerged during the pandemic. Individuals from historically excluded communities (eg, Black and Latin American) experience disproportionately negative health outcomes related to COVID-19. Community gaps in COVID-19 education, social, and health care services (including vaccines) should be prioritized as a critical effort to end the pandemic. Misinformation created by the politicization of COVID-19 and related public health measures has magnified the pandemic’s challenges, including access to health care, vaccination and testing efforts, as well as personal protective equipment. Information and Communication Technology (ICT) has been demonstrated to reduce the gaps of marginalization in education and access among communities. Chatbots are an increasingly present example of ICTs, particularly in health care and in relation to the COVID-19 pandemic.

**Objective:**

This project aimed to (1) follow an inclusive and theoretically driven design process to develop and test a COVID-19 information ICT bilingual (English and Spanish) chatbot tool named “Ana” and (2) characterize and evaluate user experiences of these innovative technologies.

**Methods:**

Ana was developed following a multitheoretical framework, and the project team was comprised of public health experts, behavioral scientists, community members, and medical team. A total of 7 iterations of ß chatbots were tested, and a total of 22 ß testers participated in this process. Content was curated primarily to provide users with factual answers to common questions about COVID-19. To ensure relevance of the content, topics were driven by community concerns and questions, as ascertained through research. Ana’s repository of educational content was based on national and international organizations as well as interdisciplinary experts. In the context of this development and pilot project, we identified an evaluation framework to explore reach, engagement, and satisfaction.

**Results:**

A total of 626 community members used Ana from August 2021 to March 2022. Among those participants, 346 used the English version, with an average of 43 users per month; and 280 participants used the Spanish version, with an average of 40 users monthly. Across all users, 63.87% (n=221) of English users and 22.14% (n=62) of Spanish users returned to use Ana at least once; 18.49% (n=64) among the English version users and 18.57% (n=52) among the Spanish version users reported their ranking. Positive ranking comprised the “smiley” and “loved” emojis, and negative ranking comprised the “neutral,” “sad,” and “mad” emojis. When comparing negative and positive experiences, the latter was higher across Ana’s platforms (English: n=41, 64.06%; Spanish: n=41, 77.35%) versus the former (English: n=23, 35.93%; Spanish: n=12, 22.64%).

**Conclusions:**

This pilot project demonstrated the feasibility and capacity of an innovative ICT to share COVID-19 information within diverse communities. Creating a chatbot like Ana with bilingual content contributed to an equitable approach to address the lack of accessible COVID-19–related information.

## Introduction

### Disparities and COVID-19

During the pandemic, disparities in COVID-19 information and vaccine uptake have emerged within the context of the broader public health crisis. Individuals from historically excluded communities, particularly Black and Latin American, experience disproportionately negative health outcomes related to COVID-19, including increased incidence, hospitalization, and mortality [1]. Although vaccination against COVID-19 is the most effective method to prevent severe symptoms, hospitalization, and death, disparities persist in vaccine intention and uptake based on race, ethnicity, language, household income, political ideology, and other factors [2,3].

Medical systems and providers often represent structural biases that perpetuate disparities for historically excluded communities [4]. Public trust in health care has significantly eroded over the past 50 years as a result of managed care, financial incentives, social media, and growing channels of misinformation [5]. This gap in trust is even more pronounced for those in historically excluded communities who have an expectation of receiving a lower quality of care resulting from a long history of health disparities in the United States [6-8]. Indeed, previous work has demonstrated that 47% of those with COVID-19 vaccine concerns for children with disabilities did not trust the medical system [9].

Communities with more significant pandemic-related risks are typically marginalized, and the lack of equitable access to information about COVID-19 is another layer exacerbating preexisting sociostructural determinants of health [10]. Misinformation, created by the politicization of COVID-19 and related public health measures, has magnified the pandemic’s challenges, including access to health care, vaccination and testing efforts, as well as personal protective equipment [11]. Of note, disparities are perpetuated by factors beyond the individual choices or preferences of community members. Predictors of COVID-19–related outcomes include societal structural features; for example, both racial residential segregation and income inequality are strong contributors to infection and mortality [12,13].

The onset of the COVID-19 pandemic with its sudden spread and high mortality rate, political polarization, intensified distrust, and misinformation, alongside public health messaging struggling to keep pace with evolving medical knowledge[14], created “a perfect storm” of confusion and concern, especially as COVID-19 vaccines were developed, tested, and rolled out to the public.

### Technology-Based Solutions

Within the context of medical systems that are perceived as untrustworthy, creative solutions that disseminate evidence-based information through other modalities are strongly needed. Information and Communication Technology (ICT) has demonstrably reduced marginalization gaps in education and access among communities globally [14,15]. ICT’s usefulness in daily life and long-term adoption is partially driven by preferences and perspectives of representative community members [16].

Chatbots are an increasingly present example of ICTs, particularly in health care and in relation to the COVID-19 pandemic [17]; these interactive software programs communicate with users through voice or text. Health care chatbots are designed under specific frameworks that contain interconnected layers, including knowledge (ie, database content), service (ie, decision-making process), dialogue (ie, prompts and responses relayed to the user), and presentation (ie, interface that communicates responses to the user via text or voice) [18]. Although there is limited research on the effectiveness of chatbots in health care [19], ICTs generally have many benefits for health care decision-making and referrals.

Because they can be used on smartphones and other mobile devices, chatbots are more accessible than traditional forms of health communication, especially in underresourced communities. This format also aligns with an increasing preference to engage with information digitally and with accessibility features for nonnative English speakers and those with disabilities. They also may help with historical mistrust of health care systems, as they are generally seen independent of and more neutral than medical providers. Overall, chatbots represent a powerful opportunity to share vital real-time, evidence-based information about COVID-19 vaccines in an environment where the virus and treatments continue to rapidly evolve.

### The Current Project

Previous research on vaccine perceptions and concerns in New York State identified several barriers to vaccine access, including mistrust of medical systems, misinformation about vaccine effects, and lack of access to the vaccine itself [9,20]. To address regional and community-level gaps in COVID-19 services and education and to promote an interface that better spreads accessible information, an ICT chatbot tool named “Ana” was developed by our team. This project included the development, implementation, and preliminary evaluation of Ana within New York State (primarily western New York). We aimed to follow an inclusive and theoretically driven design process that develops, pretests, and tests a COVID-19 ICT and evaluate user experiences. This paper describes the development and refinement process of Ana and initial data on user satisfaction; it also characterizes users as related to their engagement with Ana and their most common vaccine-related concerns.

## Methods

### Project Design

**Figure 1:**
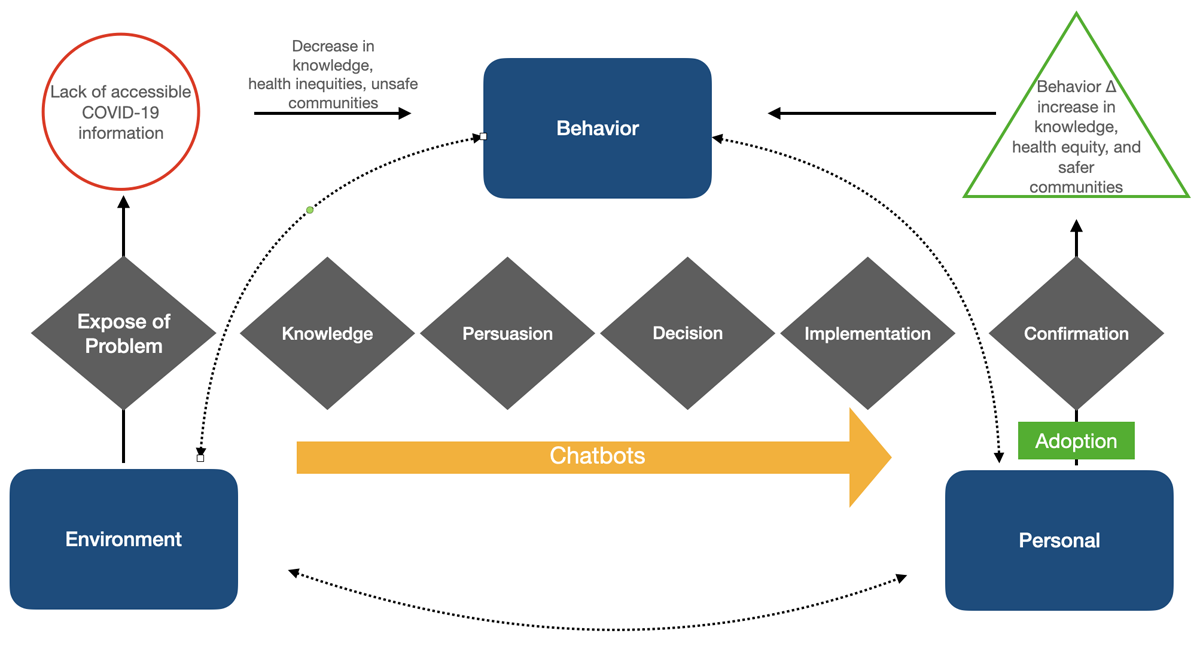
Chatbot multitheoretical framework process.

The development of Ana the chatbot followed an Agile project management methodology using a multitheoretical framework that integrates Diffusion of Innovations Theory and Social Cognitive Theory seen in Figure 1 [21,22]. The multitheoretical framework conceptualizes the information spread of a specific population or social system, expecting that people will adopt a new idea, behavior, or product. This learning occurs in a social context with reciprocal interactions of the person, environment, and their behaviors. With an emphasis on “social influence, and external and internal social reinforcement,” they experience the idea, product, or behavior as something new [23]. The framework addresses the aims of the pilot project to assess how information is disseminated through Ana to influence behavior changes in communities.

Ana was built in response to previously identified community needs of people with developmental disabilities and their families [9,20]. The studies highlighted the need of clear and concise information that would directly address the concerns and frequent questions of the community. Concerns included vaccine safety and a lack of an identified trustable source of information. The multitheoretical framework addresses the need for behavioral changes that combat both mis- and disinformation using plain, community-adapted language while guiding the design, implementation, and adoption of Ana.

### Pretesting and Refinement

The project team was multidisciplinary and diverse, including medical and public health experts, behavioral scientists, and community members. Throughout the development process, 7 iterations of Ana were presented and tested in English by longstanding national and local community partners and stakeholders. The Spanish version was also created, pretested, and culturally adapted with Spanish-speaking community stakeholders. The intention behind using multiple iterations was responsivity for expert and community feedback on content, language, and user experience (UX). The process for soliciting feedback included sending Ana versions to the abovementioned stakeholders with a REDCap (Research Electronic Data Capture; Vanderbilt University) survey link for feedback. The survey was designed to elicit experiences of using the chatbot on smartphones and computer devices, including questions about usability, barriers, accessibility, literacy, and accuracy of the content. Study personnel was notified when a new survey was completed, facilitating synchronous revisions to the chatbot that was event based. Most revisions based on this feedback were related to UX faulty chat mechanisms (ie, broken links) and making the language more accessible.

A second part of the development process included disseminating the same survey to solicit feedback from additional testers and as a convenience sample, including 22 fellows from the Leadership Education in Neurodevelopmental and Related Disabilities composed of physicians, psychologists, social workers, public health experts, audiologists, speech and language pathologists, dentists, residents, community advocates, and medical students. This iterative process, including multiple stakeholders ensured validation of Ana’s alignment with community perspectives and priorities. Ana was launched during August (English) and September (Spanish) of 2021 using snowball sampling and a series of community events [24].

### Developing Software Strategies

Ana is a cross-platform mobile and web-based app on the Landbot Chat Platform—a platform that allows developers to create rule-based and artificial intelligence bots. This enabled specific customization, such as the inclusion of graphic interchange format images (GIFs), which are an effective mechanism to improve mental health, especially during discussions and dissemination of potentially stressful or conflicting topics [25]. Google Firebase was used to build a database of chatbot variables data transferred from the Landbot platform for analyses.

### Content Creation, Management, and UX

Ana’s content was curated primarily to provide users with factual answers to common questions about COVID-19, including vaccine and children, fertility and pregnancy, vaccine options, vaccine boosters, vaccine side effects, virus variants, public health guidelines, and resources. To ensure content relevance, topics were driven by community concerns and questions [3,9,20] as ascertained through research (eg, surveys) and outreach (eg, virtual question and answer and town halls). Ana’s repository of educational content was based on national and international organizations (eg, the World Health Organization and Centers for Disease Control and Prevention) as well as interdisciplinary experts from the University of Rochester School of Medicine and Dentistry network in medicine, psychology, and public health. Ana’s users were able to engage directly with the New York State Department of Public Health COVID-19 Vaccine Hotline to schedule vaccine appointments, identify testing sites, and explore the prevalence of the virus in their geographical locations. Ana’s content was regularly updated accordingly to new guidelines and information by the project team.

### Dissemination

**Figure 2:**
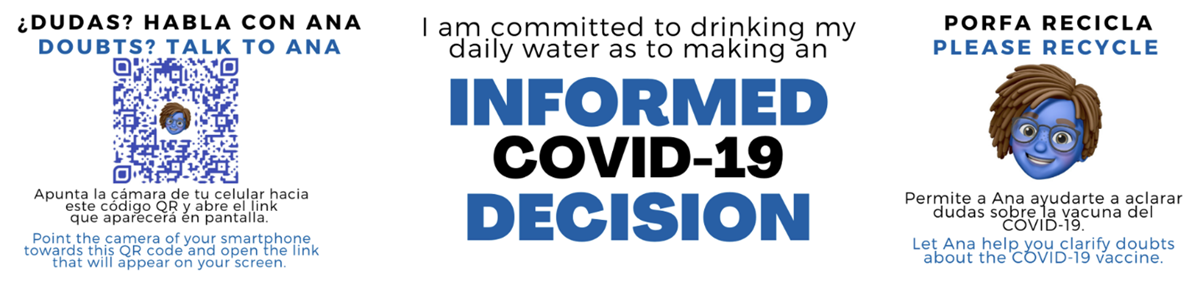
Community dissemination material—water bottle label.

As part of a community partnership effort, community members, including social and medical organizations in New York State, were actively engaged in the project. The dissemination period was between August 2021 and March 2022. Ana was disseminated in pediatric behavior and developmental disabilities–focused organizations, health fairs, social media promotional content (via Instagram, Twitter, and Facebook), social events, and other health-related offices as a process to facilitate buy-in and adoption (more detail is provided in “Pretesting and Refinement” section). Community listserves were used to share Ana with local and statewide provider agencies and advocacy groups. Bilingual (English and Spanish) promotional materials were created, including flyers (mainly for clinical offices) and water bottles (distributed during community events) with QR codes (Figure 2).

### Evaluation Framework

**Figure 3:**
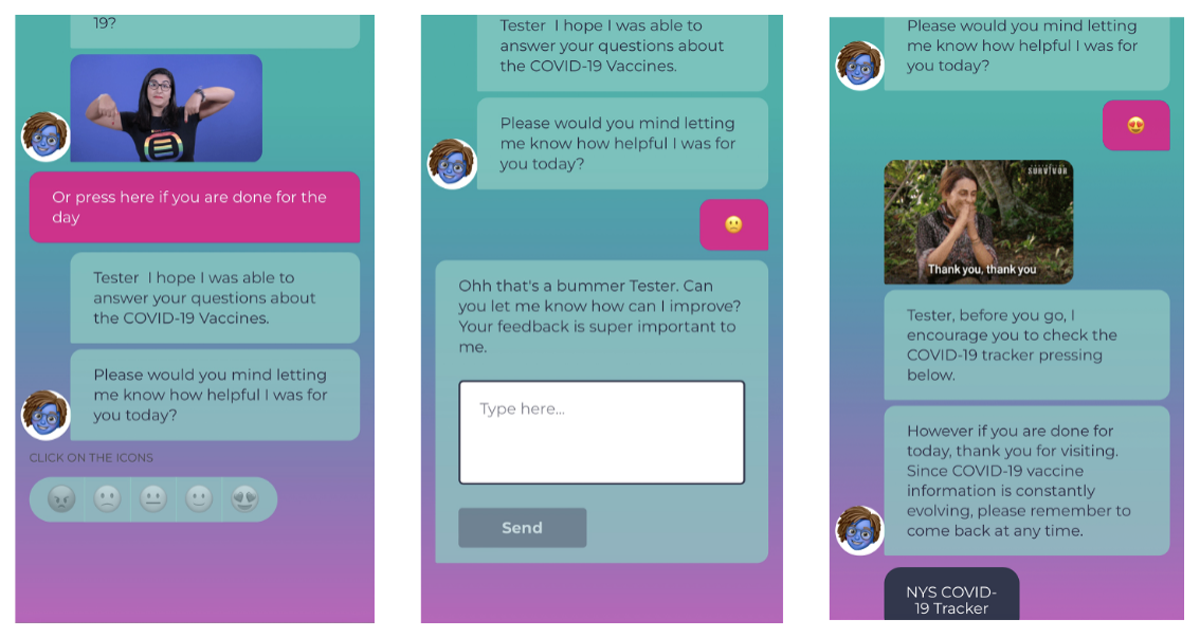
User experience satisfaction process.

In the context of the pilot project, we identified an evaluation framework to explore reach, engagement, and satisfaction. Reach included the total number of users and distribution of users across Spanish and English versions of Ana. We also aimed to characterize English and Spanish users by the content they accessed. Engagement measures included number and percentage of return users (ie, those who used Ana more than once), number of requests for vaccine information through the app, and the number of users who used Ana to directly contact the local COVID-19 vaccine hotline. To measure satisfaction (inspired by McIntosh et al [26] and Alismail et al [27]), at the end of each interaction with the chatbot, users were asked to rate their experiences using a 5-point emoji system ranging from a mad face to happy faces (Figure 3). For analysis purposes, the emojis were categorized as binary variables, wherein mad, sad, and indifferent faces prompted users to leave feedback (qualitative open-ended responses boxes), and happy faces thanked users for their feedback and invited them to share the chatbot with others. Using free-text response options, additional information was collected on both vaccine-related concerns or questions and qualitative feedback on UX.

### Ethical Considerations

Our team’s previous projects, which contributed to informing this project, were determined by the University of Rochester Institutional Review Board as “nonresearch”; and based on the United States Federal Regulation 45 CFR 46 for the protection of human subjects, “Common Rule,” this project did not meet the categories for the needs of the Institutional Review Board review process [9,20]. However, thoughtful of the importance of ethical considerations, this project was presented at the University of Rochester Medical Center Health Lab (a technology and innovations-driven think tank multidisciplinary hub) and approved by the Technology Advisory Council of the University of Rochester Medical Center as part of an internal regulatory process.

### Positionality Statement and Ethical Implications

As part of a continuing process to reflect on equity in research and inspired by the work of O'Leary et al [28] and Unertl et al [29], we acknowledge that ethical implications naturally exist while conducting academic work within the community, particularly if the work is being conducted with communities typically excluded and marginalized. The intent of this project is to facilitate health access during the COVID-19 pandemic to factual information for the community, which includes most often minoritized groups. Our team, although we acknowledge personal biases and systemic levels of academic privileges, is composed of a majority of people that come from minoritized and historically excluded groups (eg, Latines, Black, Brown, and deaf). Having a diverse and multiethnic team created a persistent dynamic “check and balances” environment helping to reflect accountability and respect for the information provided to the community inside and outside of academia. This process helped to reduce potential biases and facilitate a culture of ethical responsibility for the use of technology.

## Results

### Reach

A total of 626 community members used Ana during the time frame of August 2021 to March 2022. Among those participants, 346 used the English version with an average of 43 users per month and 280 participants used the Spanish version with an average of 40 users monthly.

#### English Version User Characteristics

**Figure 4:**
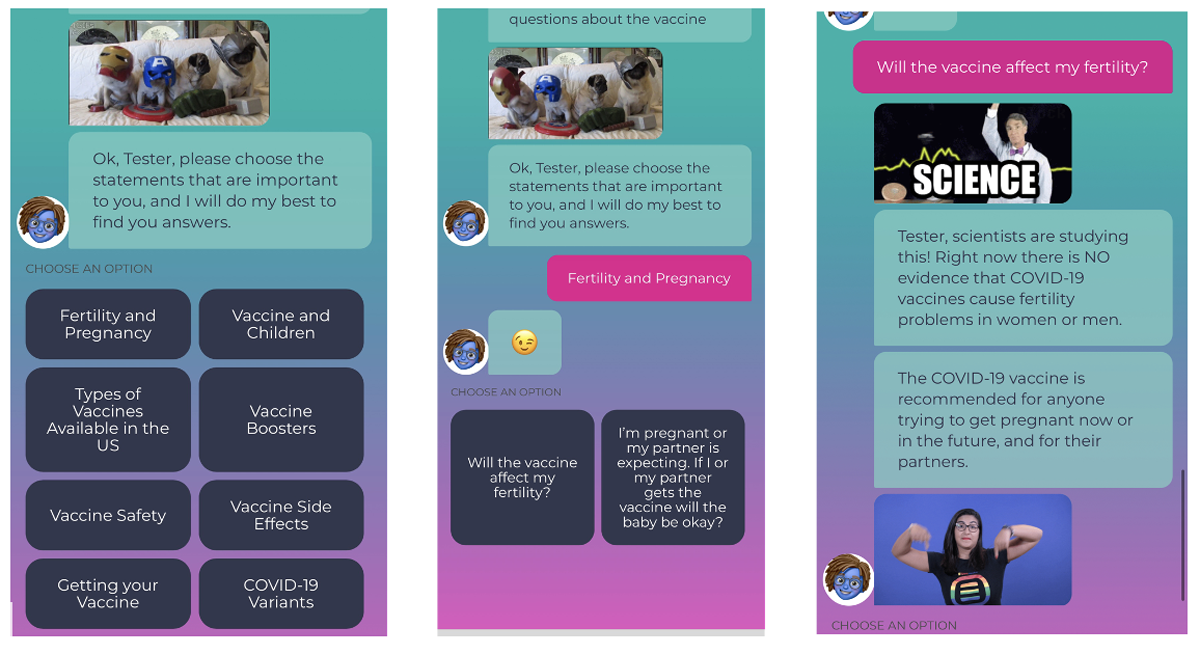
English users screen interactions.

Per Table 1, English language users were interested primarily in answering questions related to vaccination among children, vaccine and pregnancy, and safety in general (Figure 4). Among English users, 57.80% (200/346) selected the question “When will younger children be able to get vaccinated?” and 53.75% (186/346) selected the questions about if the COVID-19 vaccine could affect their child development. In addition, 20.23% (70/346) of users selected the question “Is the COVID-19 Vaccine an experiment?”

**Table 1:** English users most common interactions topics.

Items	Values, n (%)
When will younger children be able to get vaccinated?	200 (57.80)
Will the vaccine affect my child development?	186 (53.75)
Vaccine is safe for pregnancy and baby	150 (43.33)
Vaccine safety	115 (33.23)
Used the option to call the NYSDOH^a^ directly	134 (38.72)
How to identify real factual information versus misinformation	88 (25.43)
Is the COVID-19 vaccine an experiment?	70 (20.23)

^a^NYSDOH: New York State Department of Health.

#### Spanish Version User Characteristics

**Figure 5:**
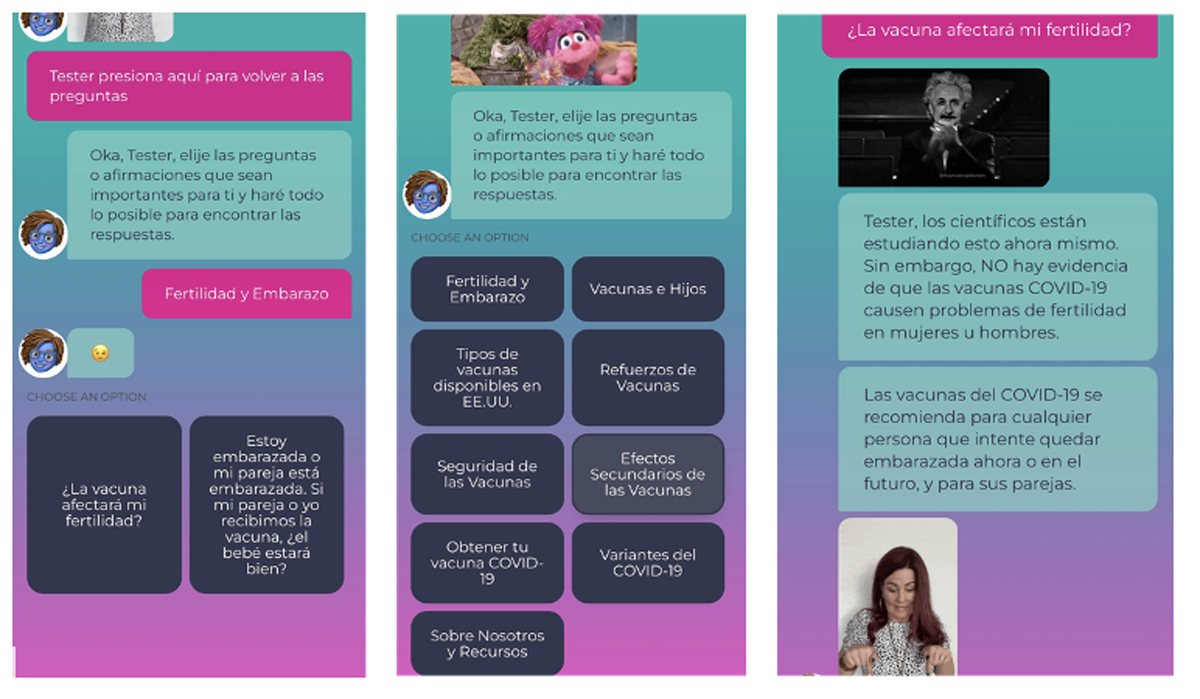
Spanish users screen interactions.

As Table 2 shows, similarly to the English version users, the Spanish version users expressed interests in topics related to vaccine and pregnancy, vaccine safety, and vaccine and children (Figure 5). For example, 66.78% (187/280) users seeked information about fertility “la vacuna afectará mi fertilidad” (the vaccine will affect my fertility), and 7.14% (20/280) users looked for more information about “seguridad de las vacunas” (vaccine safety). In contrast to the English version users, however, a majority of Ana’s users in Spanish (183/280, 65.35%) were interested in learning more about COVID-19 resources and chatbot content references.

**Table 2:** Spanish users most common interactions topics.

Items	Values, n (%)
Will the vaccine affect my child development?	187 (66.78)
References and resources	183 (65.35)
Used the option to call the NYSDOH^a^ directly	105 (37.50)
Vaccines and children	20 (7.14)
Vaccine safety	20 (7.14)

^a^NYSDOH: New York State Department of Health.

### Engagement

**Figure 6:**
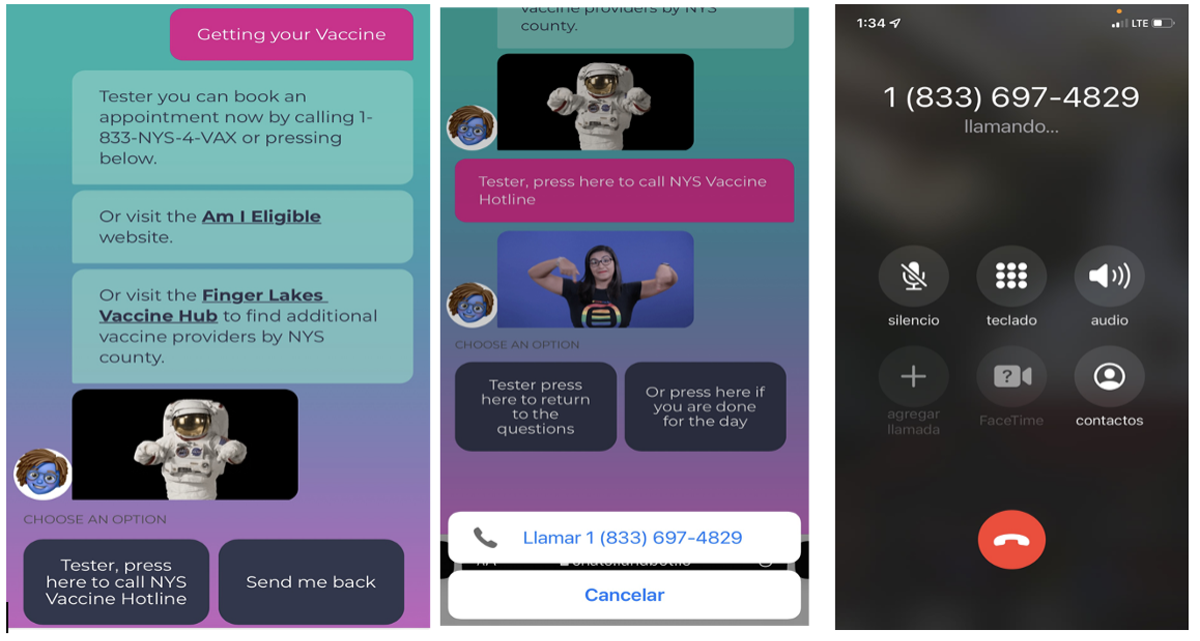
New York State COVID-19 vaccination hotline feature.

Across all users, 63.87% (221/346) of English users and 22.14% (62/280) of Spanish users returned to use Ana at least once. The month of November had the highest frequency of users among both Ana’s platforms (English: n=127; Spanish: n=133), respectively. Among users, a total of 19.94% (69/346) of English users and 7.85% (22/280) of Spanish users expressed interest in receiving the vaccine booster information. The majority of the users requesting vaccine booster information were vaccinated (English version users: 49/69, 71.0%; Spanish version users: 133/133, 100%) with either two shots from Pfizer-BioNTech and Moderna or Johnson and Johnson (Janssen) vaccines. In addition, 21.72% (5/22) of Spanish version users and 20.23% (14/69) of English version users reported to have received at least one booster shot series. As part of Ana’s built-in infrastructure, users could select an option to call directly the New York State Department of Health COVID-19 Vaccination Hotline to answer further questions related to the vaccine and to schedule a vaccine appointment (Figure 6). As a common behavior among users in both platforms, a majority of users (English: 134/346, 38.72%; Spanish: 105/280, 37.50%) used this feature.

### Satisfaction

#### Quantitative Feedback

**Figure 7:**
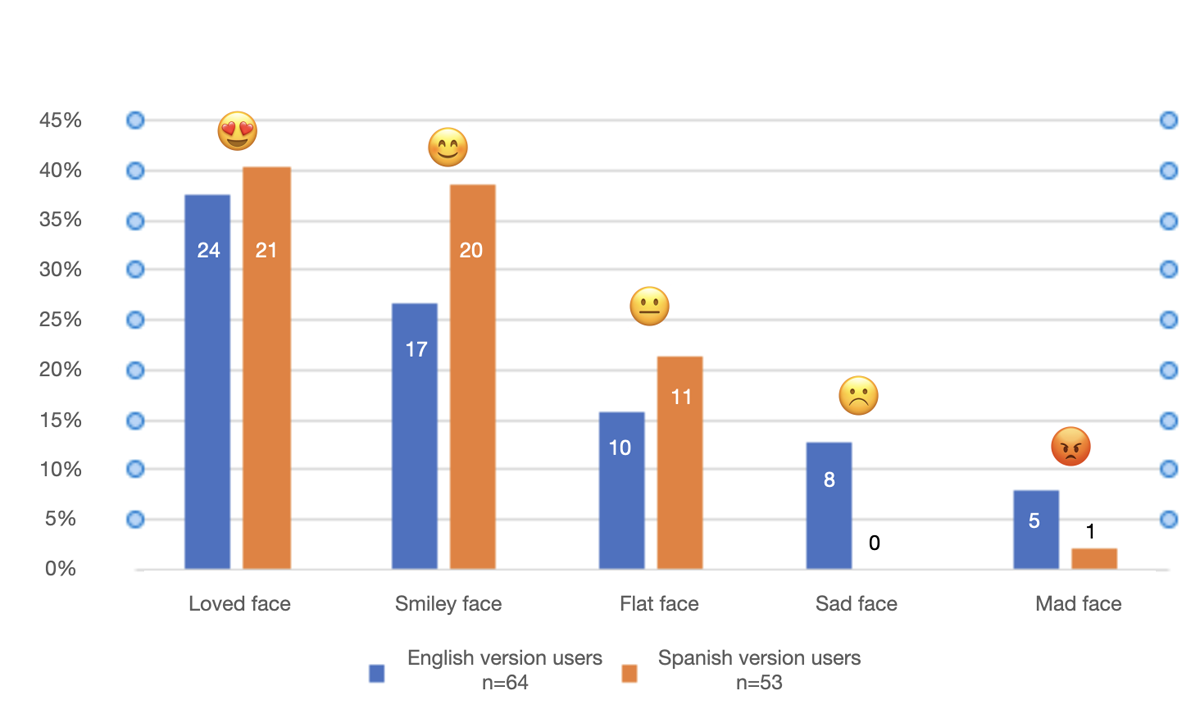
Ana’s user experience rating.

A total of 18.49% (64/346) of English users and 18.57% (52/280) of Spanish users reported their feedback (Figure 7). UX ranking categories were combined into binary variables (positive vs negative). Positive ranking comprised the “smiley” and “loved” emojis, and negative ranking comprised the “neutral,” “sad,” and “mad” emojis. When comparing negative and positive experiences, positive experiences were higher across Ana’s platforms (English: 41/64, 64.06%; Spanish: 41/53, 77.35%) versus negative experiences (English: 23/64, 35.93%; Spanish: 12/53, 22.64%).

#### Qualitative Feedback

Qualitative feedback (Textbox 1) was solicited from users who reported neutral and unsatisfactory experiences. UX feedback was organized around two major themes, as follows: (1) disagreement with or mistrust of the content, as expressed through quotes such as “The vaccines are made for suckers” and “No le veo mucha utilidad” (this is useless); and (2) suggested changes to chatbot usability, including reducing the speed flow of the chat answers (eg, “Slow down, you talk too fast”) and the limited language access of some multimedia, such as informative videos (eg, “Algunos videos no están en español” [some of the videos are not in Spanish]).

**User experience qualitative feedback.**

English

- “Horrible experience. Biased information.”
- “The vaccines are made for suckers.”
- “NO GOOD”
- “This is bs.”
- “I would like to know from where are you getting the information posted here?”
- “Slow down! You talk too fast!”
- “Info on children age for vaccine is not up to date.”
- “Some memes are not interesting.”

Spanish

- “Mucha información falsa” (too much misinformation).
- “Es una babosada llena de politicas liberales” (it is silliness full of liberal politics).
- “No le veo mucha utilidad” (I do not see much utility for it).
- “A veces la información pasa muy rápido” (sometimes the information goes by too fast).
- “Algunos videos no están en español” (some videos are not in Spanish).
- “Los memes son gracioso, pero el covid no lo es” (memes are funny, but COVID is not).

## Discussion

### Principal Findings

This pilot project demonstrated the feasibility and capacity of an innovative ICT to share COVID-19 information within diverse communities. Creating a chatbot (Ana) with content in both English and Spanish languages contributed to an equitable approach to address the lack of accessible COVID-19–related information, particularly among minoritized groups, such as Latines and people with disabilities, which have been previously reported as groups most affected by the pandemic [30,31]. Ana’s feasibility was demonstrated through reach, engagement, and satisfaction. A high number of users were reached across the relatively brief time frame for rollout, and there was fairly equal representation of both English- and Spanish-speaking users. With respect to engagement, Ana may have been successful in connecting many users to additional resources, given that almost 40% (English: 134/346, 38.72%; Spanish: 105/280, 37.50%) of individuals used Ana to call the COVID-19 vaccine hotline. Return engagement was somewhat higher for English content, with almost two-thirds using Ana more than once, as compared to about one-fifth of users for Spanish content. It may be that building trust with Spanish-speaking users took longer than with English-speaking users. These differences did not appear to be driven by satisfaction, as ratings of UXs were comparable for both English and Spanish content (Figure 7). Overall, the satisfaction ratings aligned with commonly observed metrics for other chatbots, including those used in health care [32,33].

Using the chatbot Ana was fairly consistent across both languages and month to month (approximately 80 users per month), with the exception of high traffic in November 2021. This may be explained by multiple factors, including the approval for the COVID-19 Pfizer-BioNTech vaccines among children 5 to 11 years of age; the Food and Drug Administration approval of Paxlovid, the first antiviral in-home treatment for high risk for COVID-19 severe illness [34]; and studies from the Centers for Disease Control and Prevention presenting the disproportionate hospitalization and mortality risk for unvaccinated individuals [35]. Furthermore, COVID-19 cases and hospitalizations increased in November across the New York State area with approximately 5000 daily cases [36].

We characterized the topics and concerns most commonly selected across user groups. Significant overlap was observed with respect to concerns about vaccine safety as well as topics related to children and vaccines (eg, safety in children, effects on child development, and safety in pregnancy). These patterns align with our previous community research on vaccine concerns as well as those documented in national surveys [3,20,37]. The connection between Ana topics and community concerns was in itself a positive outcome and an intentional aim of the project. We view this alignment as Ana being a good *fit* for our community, which was likely influenced by our explicit community engagement throughout the development process.

Although satisfaction of Ana was generally high, negative feedback was shared through ratings and free text. Some unsatisfied users suggested changes to the user interface, which will be helpful in informing future refinements for Ana. Other negative feedback centered around perceptions of the content being biased, misinformed, and politically liberal. Clearly, receiving information from a neutral source, such as a chatbot, is not sufficient to engender trust with all users, nor did we expect this to be the case. Factors such as political ideology and general mistrust in institutional systems (eg, government and medical systems) interact with each other to reduce intention to receive COVID-19 vaccination [38]. Further, historical trauma within medical systems has created significant barriers to faith in vaccines, particularly for Black and Indigenous communities, people of color [39], and people with disabilities [40]. These are complex constructs that will not be sufficiently addressed simply through ICT use. Rather, the intention for Ana is that its use will increase equity in access to information about the COVID-19 vaccine, which may be an important initial step in the decision-making process.

To this point, our current data on Ana reflect the initial stages of the multitheoretical framework process. Namely, users acquired knowledge from Ana, which may have impacted their persuasion to get inoculated and practice safer behaviors to reduce COVID-19 exposure. Users’ engagement with these initial stages may influence their actual decision-making, but we did not track the connection of chatbot use to actual vaccination behaviors. Additionally, satisfaction data can provide an initial proxy for adoption, although further dissemination and data on reach are needed to fully capture true adoption.

### Limitations and Implications for Future Projects

Given our focus on addressing health equity, we note some limitations to consider for achieving greater impact of ICTs. Ana was available in only 2 languages, and some of the videos shared by Ana were only available in English, which presents a limitation to language access. Despite the fairly high engagement (626 participants in 7 months), there was a low rate of UX ratings; as such, feedback may not be fully representative of all Ana users. Furthermore, qualitative feedback on UX was collected only from those who gave negative quantitative ratings (ie, selected the negative emoji categories). Accordingly, the qualitative feedback was negatively skewed. Direct solicitation of open-ended feedback from those who had positive experiences with Ana will be important for a more robust assessment of user satisfaction. Additionally, although the project team attempted to use the lowest chat speed response ratio, users reported Ana’s chatting speed between messages to be a challenge; this may not be addressable using the current platform and may suggest a need to explore alternatives.

Despite the fact that this pilot project presents some limitations, there are some lessons learned that could impact the development and implementation of ICTs, such as chatbots, prospectively. Although Spanish is the most spoken non-English language in the United States [41], the demographics of the community should be reviewed to consider other languages that may represent a more equitable approach to minoritized groups (eg, the deaf, refugees, or migrant workers). Ideally, the visual aids and complementary multimedia information would be available in those specific languages, with content adjusted to the corresponding culture. Qualitative positive feedback should be sought to capture insight such as “what has been helpful, why it has been valuable, and to whom (Spanish vs English speakers).” In addition, previous studies have suggested that sustainability among ICTs development and dissemination requires community engagement in the early phases [14,26]. As part of the UX with ICTs, such as chatbots, we encourage the use of short answers using simple language and a maximum of 2 monitor speed of responses and adjusting it accordingly to the answer’s length. Finally, although this project benefited from a multisectorial community stakeholder engagement, it is important to acknowledge that there is still a digital health access gap among communities, particularly for rural and marginalized groups. This gap creates a nonequitable environment that impedes public health education and hinders the use of emerging and innovative technology designed to mitigate the COVID-19 impact.

### Conclusions

Previous research demonstrates how using ICT to provide factual and real information during emergencies is essential, and this is particularly true during the COVID-19 pandemic. The lack of verifiable and trustworthy information can generate contradictory societal behaviors, perpetuating and exacerbating the negative impact of public health emergencies.

Chatbots are one viable option for closing this gap and promoting community equity. In this pilot project, we demonstrated that Ana served as an important tool to improve COVID-19 information knowledge and access for both English and Spanish speakers. With ongoing refinements and dissemination, we hope that Ana and similar technologies will effectively address health disparities among marginalized communities and contribute to ending the pandemic.

## Abbreviations

ICT: Information and Communication Technology
UX: user experience

## Data Availability

The data sets (excluding chatbot coding) generated and analyzed during this study are available from the corresponding author upon reasonable request.
